# Molecular Serotyping and Antibiotic Resistance Patterns of *Escherichia coli* Isolated in Hospital Catering Service in Morocco

**DOI:** 10.1155/2020/5961521

**Published:** 2020-08-19

**Authors:** Benjelloun Touimi Ghita, Laila Bennani, Sanae Berrada, Moussa Benboubker, Bahia Bennani

**Affiliations:** ^1^Laboratory of Human Pathology Biomedicine and Environment, Faculty of Medicine and Pharmacy of Fez (FMPF), Sidi Mohammed Ben Abdellah University (USMBA), Fez, Morocco; ^2^Faculty of Sciences and Techniques of Fez (FSTF), USMBA, Fez, Morocco; ^3^The Superior Institute of Nursing Professions and Health Technology of Fez (ISPITS), Fez, Morocco; ^4^Nursing Department, Hassan II University Hospital, Fez, Morocco

## Abstract

*Escherichia coli* is related to foodborne disease and outbreaks worldwide. It mainly affects persons at high risk as newborns, infants, and individuals with impaired immune system in hospitals. Multidrug-resistant *E. coli* is currently spreading both in community and hospital settings. Our study aims to evaluate the presence of *E. coli* and the incidence of its antibiotic resistance in samples obtained from various cooked and raw foods (*N* = 300), food contact surfaces (*N* = 238), and food handlers (*N* = 40) in Moroccan hospital catering service. *E. coli* was identified using API 20E, and the antibiotic resistance patterns were obtained using the agar disk diffusion methods. However, PCR method was used for O157 and H7 typing. The samples analysis showed that 14.33%, 24.16%, and 45% of food, surfaces, and food handlers harbored *E. coli*, respectively, with the highest rates obtained in raw meats (34.88%) and salads (34.88%). Molecular amplification shows that 14 *E. coli* isolates carried the flagellar antigen H7, while there are no isolates showing amplification for O157. The high rate of resistance was noted against ampicillin (100%), amoxicillin-clavulanate acid (100%), nalidixic acid (61.62%), and cefotaxime (59.49%), and isolates obtained from food handler's hands showed the highest rates of resistance. None of the isolates are extended-spectrum beta-lactamases producing, while 27.7% of the isolates were metallo-beta-lactams producing. This first study conducted on Moroccan hospital catering services may draw the authorities' attention to the necessity of setting up a surveillance system to monitor the food preparation process and the safety of prepared food in healthcare settings.

## 1. Introduction

*Escherichia coli* (*E. coli*) is facultative anaerobic, non-spore forming, Gram-negative bacteria within the Enterobacteriaceae family. It forms part of the humans and warm-blooded animals natural gastrointestinal flora [1, 2]. Although most *E. coli* are harmless commensal organisms [3], several pathogenic strains can cause a variety of diseases. There are six pathogenic groups with different virulence traits and mechanisms of pathogenicity [4]. Diarrheagenic *E. coli* O157:H7 is a common serotype [5] and the main cause of foodborne disease and outbreaks worldwide. It represents a risk to global food safety and public health [6] primarily for persons at high risk as newborns, infants, and individuals with compromised immune system [2].

Besides their pathogenesis and virulence, *E. coli* strains also acquire resistance over time. Mobile DNA elements, temperate bacteriophage, and transmissible plasmid have all served as carriers for antibiotic resistance genes in *E. coli* [7]. In fact, *E. coli* belongs to the group I enterobacteria, characterized by a phenotype naturally sensitive to all *β*-lactams. It is also naturally sensitive to other classes of antibiotics including aminoglycosides, quinolones, sulfonamides, trimethoprim, tetracycline, and chloramphenicol. However, this bacterium is a good example of antibiotic resistance evolution, and multidrug-resistant (MDR) strains are currently spreading in both community and hospital settings [8]. Due to their ubiquity and infections frequency, the spread of MDR *E. coli* is of great concern to the medical community [7]. Therefore, MDR bacteria contaminated food could be a serious threat to consumer health especially to vulnerable patients in hospitals [9, 10] who are highly disposed to infection due to their illness, treatment, or their compromised immune system [7, 11]. Hence, several epidemiological studies have shown the correlation between increased morbidity and mortality with resistant foodborne pathogens infections [12, 13]. Thus, nosocomial foodborne infections prevention is essential, and implementing a surveillance system in healthcare setting is required.

There are few data on the prevalence and antimicrobial resistance of *E. coli* in food, food surfaces, and food handlers in Morocco. This is the first study that evaluates the presence of *E. coli* and O157:H7 serotype and determines their antibiotics resistance in a Moroccan hospital kitchen environment (food, food contact surfaces, and food handlers (hand carriage)).

## 2. Materials and Methods

### 2.1. Study Design

This study was conducted in a hospital food service in Morocco that produces more than 1000 meals per day to serve patients and medical staff. *E. coli* detection was performed on food, food contact surfaces, utensils, and hand samples among food handlers.

### 2.2. Sample Collection

A total of 608 samples were collected from May 2015 till June 2016. They were 300 food samples, 238 surfaces and utensils samples, and 40 hand samples. Food samples were collected aseptically using sterile spoons and placed into Stomacher bags (Grade, UK). The targeted surfaces and an area ranging from 20 to 100 cm^2^ (according to the dimension of the surface to be sampled) were swabbed by sterile cotton swabs (Oxoid, UK), premoistened into a 2 mL sterile Brain heart Infusion solution (Oxoid, UK) then transported to the laboratory in ice boxes (4°C) [14]. The biological samples were collected from bare-hand food handlers using swabs as recommended by Evancho et al. [15]. All samples were obtained during the food preparation process.

### 2.3. *E. coli* Isolation and Identification

Twenty-five grams of each samples was homogenized with 225 mL of sterilized peptone water. 1 mL aliquots of the homogenate was inoculated into 15 mL of Violet Red Bile Lactose Agar (Biokar Diagnostics, France) then incubated for 24 h at 37°C. The presumptive colonies were streaked into eosin methylene blue agar (Biokar Diagnostics, France). *E. coli* identification was based on morphological, microscopic characteristics and biochemical assays, including gram straining, oxidase, indole, fermentation, citrate degradation, then confirmed by API 20E kit (BioMerieux, France).

### 2.4. Molecular Detection of *fliCH7* and *rbfO157* Genes in the Isolated Strains

Molecular serotyping was performed on all *E. coli* isolates using PCR to detect the serogroup O157 and the H7 antigen (Table 1). The obtained PCR products were sequenced using an ABI PRISM 3130DNA Analyzer and the data were processed with the Sequencing Analysis 3.3 software (Applied Biosystems).

### 2.5. Antimicrobial Susceptibility Testing

Antimicrobial susceptibility was determined using disk diffusion method on Muller–Hinton agar (Biokar Diagnostics, France) and interpreted according to the European Committee on Antimicrobial Susceptibility recommendations [18]. The tested antibiotics (Oxoid, UK) were ceftazidime (30 *μ*g), cefotaxime (30 *μ*g), trimethoprim-sulfamethoxazole (25 *μ*g), gentamicin (30 *μ*g), ciprofloxacin (30 *μ*g), amikacin (30 *μ*g), ampicillin (10 *µ*g), amoxicillin-clavulanic acid (20/10 *µ*g), and imipenem (10 *μ*g).

### 2.6. Phenotypic Detection of Extended-Spectrum Beta-Lactamases (ESBL) and Metallo-Beta-Lactamases (MBL)

ESBL presence was checked by double-disk synergy test. It was performed on agar with 30 *µ*g disk of cefotaxime, ceftazidime and a disk of amoxicillin-clavulanate (20 *µ*g/10 *µ*g) positioned at a distance of 30 mm. The test was considered positive when a decreased susceptibility to cefotaxime combined with a clear-cut enhancement of the inhibition zone in front of the clavulanate-containing disk was observed [19].

EDTA disk synergy test was used to detect metallo-beta-lactamases (MBL) activity. A suspension of *E. coli* (0.5 McFarland) was swabbed over Mueller–Hinton agar (Biokar Diagnostics, France) surface. After drying, an imipenem disk (10 *µ*g) (Oxoid, UK) and a blank filter paper disk were placed 10 mm apart from edge to edge; then 10 *µ*L of 0.5 M EDTA solution was added to the blank disk. After overnight incubation, the presence of an expanded inhibition zone was interpreted as EDTA synergy test positive [20].

## 3. Results

### 3.1. *E. coli* Prevalence

The samples' bacteriological analysis shows that 14.33% of food samples harbored *E. coli* (Table 2). The highest rates were isolated in raw meats (34.88%) and salads (34.88%), while the lowest rates were obtained in the hot meals (9.31%) and pastries. Regarding the utensils and food contact surfaces, the raw meats worktops were the most contaminated with a rate of 59.09%, whereas the pastries worktops were exempts from *E. coli*. However, *E. coli* was detected in 45% of the examined food handler's samples.

### 3.2. *E. coli* O157 H7 Molecular Serotyping

Molecular amplification shows that 14 *E. coli* isolates carried the flagellar antigen H7. However, no isolates show amplification for O157. Thus, haemorrhagic *E. coli* O157:H7 was not detected in any of the examined samples (Table 3).

### 3.3. Antibiotic and Resistance Profiles of Isolated Strains

Overall, the highest resistance rates were shown against ampicillin and amoxicillin-clavulanate acid (100%), nalidixic acid (61.62%), and cefotaxime (59.49%). As reported in Table 4, the isolates obtained from food handler's hands showed the highest numbers of resistance among all the other strains. From food and food contact surfaces samples, the highest rates of resistance were observed in isolates obtained from raw meats and sinks, respectively. The ESBL-producing *E. coli* detection was performed on isolates with decreased resistance to third-generation cephalosporins. The results show that none of the tested isolates produced an extended-spectrum *β*-lactamase (ESBL). However, 27.7% of the isolates were carbapenemase producing (MBL).

As reported in Table 5, the *E. coli* isolated strains show a high resistance to the most tested antibiotics. A total of 44 multiresistance profiles were determined. For food samples, 28 profiles were determined with a predominance of the profile “AMC. AMP. CN. SXT. NA. IPM. CAZ” resistants, while the food contact surfaces harbored 7 profiles with a predominance of the profile “AMC. AMP. CN. SXT. NA. IPM. CAZ. CFM” resistants. Additionally, 28 profiles were detected in the staff hands with a predominance of the profile “AMC. AMP. CN. SXT. NA. IPM. CAZ. CFM” resistants.

## 4. Discussion

*E. coli* is foodborne pathogen identified in food products, human, and environmental samples worldwide [2, 21]. Several studies showed and documented an increase in its drug resistance during the last decades [22–24]. The aim of the present study is to provide an informative basis on the *E. coli* prevalence and its antibiotic resistance profiles in food (raw and cooked), food contact surfaces and its carriage among food handlers in hospital catering service in Morocco. As far as we know, the present investigation is the first one conducted on a hospital catering service in this country.

The microbiological analysis demonstrated that 14.33% of food samples harbored *E. coli*. This rate is higher than the one found in Iranian hospital (6.71%) [10]. This high prevalence could be related to the long-process food production, the inadequate washing of raw materials, inadequate cooking process, and the nonrespect of the freezing/cooling temperatures [9, 10].

The highest rates of *E. coli* were detected in raw meats (34.88%) and salads (34.88%) compared to hot meals (9.31%) and pastries (4.66%). A similar tendency was reported in an Iranian study with rates of 20% and 6% in raw meats and cooked meals, respectively [10]. The high rates of raw meats harboring *E. coli* could be related to one or multiple factors: (i) the meats autocontamination during slaughter as *E. coli* is a normal part of the intestinal microflora of many animals; (ii) the inadequate hygienic conditions in slaughterhouses; (iii) the inappropriate storage temperature specially that meat is a favorable medium to rapid development of bacteria [25, 26].

The high rates of *E. coli* in salads and raw vegetables can be related to an enteric contamination before product harvesting, a nonhygienic handling process and/or a contamination during the distribution process [27].

Regarding the utensils and food contact surfaces, the raw meats cutting boards were the most contaminated with a rate of 59.09%. This rate is still lower than that reported in a Brazilian hospital (90%) [28]. In fact, several studies demonstrated that these utensils present high microbial counts due to cross-contamination [29] and are influenced by the raw nature of handled materials and the surface material type (wood, stainless steel, plastic, etc.). Hence, the presence of *E. coli* in most food surfaces can be attributed to cross-contamination between food materials and these food contact surfaces and the subsequent growth of microorganisms in biofilms [30, 31]. In fact, bacteria as *E. coli* have a strong capacity of forming biofilms which are characterized by their high resistance to antibiotics and to environmental stresses [32]. Klontz et al. described that 25% of food workers reported to reutilize cutting boards without cleaning after cutting raw chicken [33]. So, the lack of adequate food safety knowledge among food handlers [3] and the inadequate food contact surfaces cleaning and sanitizing as well as the overall sanitary conditions of food preparation in this facility can endorse the accumulation of food debris and bacteria in biofilms on surfaces.

The *E. coli* carriage among food handlers was observed in 45% of cases. Our result is similar to that of an Egyptian investigation reporting that 41% of food workers were carrying *E. coli* in their hands [34] but higher than the rates found in Malaysia (14.20%) [35]. The food service employees' hands are considered as vectors of foodborne disease spread, mainly because of poor personal hygiene. In fact, it was proved that inappropriate food handler practices contributed to approximately 97% of foodborne illnesses in food service establishments [36]. Thus, hand washing is a fundamental precautionary measure to protect against the infection spread and is one of the primary practices for reducing the bacteria transfer [37–39].

In the present study, haemorrhagic *E. coli* O157:H7 has not been detected; H7+ non-O157 strains were isolated with a low rate (2.41%). This result is similar to those reported in Moroccan study conducted on several food types (5%) [40]. However, *E. coli* non-O157 have more recently been recognized as important pathogens with an increasing impact on human health and are now also considered as major cause of disease [41]. Its virulence, arises from Shiga (Vero) toxins-production coded by Shiga toxin genes (stx1 and stx2), which are the primary factor responsible for the haemorrhagic aspect of the diarrhea and systemic complications [42]. In this study, and in spite of the limitation related to the nondetermination of Shiga toxin genes presence, the detection of *E. coli* non-O157 in the food designated to vulnerable patients constitutes a high risk. The systematic detection of this species must be recommended routinely in this setting.

Moreover, *E. coli* antibiotic resistance is a topic that is continuously reviewed, and multiple studies are published every year with new data on antimicrobial resistance genes acquisition. Even if the emergence of new strains capable of hydrolyzing new generation of cephalosporins and *β*-lactams is common among *E. coli* isolated from foods [21, 22], the antibiotic resistance of strains isolated in hospital food have not been studied widely.

In this investigation, all isolates were resistant to ampicillin and amoxicillin and highest rates of resistance were obtained against nalidixic acid (61.62%) and cefotaxime (59.49%). These rates are consistent with those reported by Ranjbar et al. in *E. coli* isolated from hospital food [9, 10]. In fact, isolates obtained from raw meats showed the highest rates of resistance to the tested antibiotics among all food sample types. This result was not surprising since a previous study conducted in Morocco shows that raw chicken harbored a high resistance against amoxicillin (90.9%) [43]. Such resistance can be related to the use of these antibiotics in livestock either as therapeutic or preventive molecules. In fact, for economic reasons, the use of those antibacterial growth promoters is permitted and leads to an increase of antimicrobial resistance risks. Knowing that *E. coli* had high ability to exchange genetic material, the use of tetracycline and ampicillin in livestock (as is the case in Morocco) helps to resist spread [44, 45].

The isolates obtained from food handler's hands showed the highest rates of resistance among all the other isolates. This result corroborates the results of studies concluding that human isolates are more resistant than food isolates [46]. Effectively, a study conducted in Qatar showed that 59% of healthy food handlers carried resistant *E. coli* in their hands and 27% were MDR [47]. The fact that healthy food handlers carry MDR *E. coli* represents an important public health risk to the general population because of the possibility of dissemination through contaminated food [47].

Independently of samples types, *E. coli* isolates show a resistance against the most tested antibiotics, and a total of 43 multiresistance profiles were determined. The profile showing resistance to “AMC, AMP, CN, SXT, NA, IPM, CAZ, CFM” was the most detected. The presence of this pattern can be explained by the large use of beta-lactams and cephalosporins as routine growth factors in veterinary and also as drugs of choice against *E. coli* in medicine [48–50].

None of the studied isolates produced an ESBL, while a large number of worldwide studies reported their important rate isolation especially from food-producing animals as chicken, beef, pork, and milk [21, 22, 51–53]. The ESBL absence is a positive point since the food is essentially destined to immune-depressed patients. Nevertheless, 27.7% of the *E. coli* isolates were carbapenemase-producing. Hence, the emergence of this isolates type in food or in the environment is worrying and an important concern for the public health sector specially that cephalosporins as “critically important” antimicrobials given that they represent the last treatment choice in human medicine for MDR bacterial infections [54, 55].

In conclusion, the present investigation is the first report on the prevalence and antibiotic resistance profiles of *E. coli* isolates obtained from food samples, food contact surfaces, and food handlers in hospital catering service in Morocco. The *E. coli* contamination was important with a high prevalence of MDR strains and the presence of *E. coli* non-O157/H7^+^. All *E. coli* isolates were resistant to at least 2 antibiotics (AMC and AMP). The obtained results highlight the need for continuous monitoring of the production chain and assessment of the implemented cleaning and sanitizing processes in order to minimize public-health risks. This study may draw the concerned authorities' attention to control the transmission of multiresistant foodborne pathogens mainly in healthcare settings.

## Figures and Tables

**Table 1:** Primers used for the detection of *flicH7* and *rfbO157* genes.

Amplified regions	Primers	Sequences (5′-3′)	Size (Pb)	Reference
*fliCH7*	Flic F	FGCGCTGTCGAGTTCTATCGAGC	625	[16]
	Flic R	CAACGGTGACTTTATCGCCATTCC		

*rfbO157*	O157 F	CGGACATCCATGTGATATGG	256	[17]
	O157 R	TTGCCTATGTACAGCTAATCC		

**Table 2:** Prevalence of *E. coli* according to samples types.

Samples	Samples collected (*N*)	Positive samples *N* (%)
*Food samples*		
Raw meats	39	15 (34.88)
Raw vegetables	33	7 (16.27)
Hot meals	150	4 (9.31)
Salads	54	15 (34.88)
Pastries	24	2 (4.66)
Total	300	43 (14.33)

*Surfaces and utensils*		
Chopping meat devise	26	12 (46.15)
Knives	10	2 (2)
Weighing machine	16	2 (12.5)
Sink	20	8 (20)
Recipient	38	3 (7.89)
Baking stainless steel worktop	30	0
Serving meals worktop	30	1 (3.33)
Raw meat cutting boards	44	26 (59.09)
Vegetables cutting boards	24	4 (16.66)
Total	238	58 (24.16)

Hand carriage	40	18 (45)

**Table 3:** Molecular serotyping results of *E. coli*.

*N* (%)		
Samples	*fliCH7*	*rfbO157*
Food (*n* = 300)	9 (3)	ND
Food contact surfaces (*n* = 208)	3 (1.25)	
Hand carriage (*n* = 40)	2 (5)	

Total	14 (2.41%)	

**Table 4:** Prevalence of antibiotic resistance.

	Samples	Prevalence of phenotypic resistance (%)								MBL		ESBL
		AMP	AMC	CN	SXT	NA	IPM	CAZ	CFM	P	N	
Food samples	Raw meats	100	100	53.3	46.7	53.3	60	53.3	60	33.3	66.7	ND
	Raw vegetables	100	100	42.9	42.9	85.7	14.3	57.1	57.1	ND		ND
	Hot meals	100	100	75	25	50	75	50	75	25	75	ND
	Salads	100	100	46.7	66.7	60	46.7	46.7	40	20	80	ND
	Pastries	100	100	50	0	100	100	0	50	ND		ND

Food contact surfaces	Chopping meat devise	100	100	58.3	50	50	58.3	50	50	25	75	ND
	Knives	100	100	100	50	50	50	100	100	ND		ND
	Weighing machine	100	100	0	0	0	0	50	50	ND		ND
	Sink	100	100	100	0	71.4	85.7	57.7	71.4	28.6	71.4	ND
	Recipient	100	100	0	100	100	100	33.3	50	ND		ND
	Baking worktops	100	100	0	0	0	0	0	0	ND		ND
	Serving meals worktops	100	100	50	50	50	75	50	100	ND		ND
	Raw meat cutting boards	100	100	69.2	46.2	65.4	50	50	50	23.1	96.1	ND
	Vegetables cutting boards	100	100	50	50	100	50	50	50	ND		ND
Hand carriage		100	100	100	55.6	88.9	77.8	94.4	88.9	38.9	61.1	ND
Total		100	100	100	35.54	61.62	51	49.5	59.49	27.7	72.3	ND

ATB: antibiotic; ND: not detected; P: positive; N: negative; AMP: ampicillin; CN: gentamicin; SXT: trimethoprim-sulfamethoxazole; NA: nalidixic acid; IPM: imipenem; CAZ: ceftazidime; CFM: cefotaxime; MBL: metallo-beta-lactamases; and ESBL: extended-spectrum beta-lactamases.

**Table 5:** Antibiotic resistance profiles detected in *E. coli*.

		Profile	*N* (%)		
			Food	Food contact surfaces	Hand carriage
2 ATB	1	AMC. AMP	-^*∗*^	—	2 (3.4)

3 ATB	2	AMC. AMP. CN	—	1 (5.6)	—
	3	AMC. AMP. NA	—	—	1 (1.7)
	4	AMC. AMP. SXT	1 (2.3)	—	—
	5	AMC. AMP. IPM	2 (4.7)	—	1 (1.7)
	6	AMC. AMP. CAZ	—	—	4 (6.9)
	7	AMC. AMP. CFM	—	—	3 (5.2)

4 ATB	8	AMC. AMP. CN. NA	1 (2.3)	—	—
	9	AMC. AMP. NA. IPM	1 (2.3)	—	—
	10	AMC. AMP. NA. CAZ	—	—	1 (1.7)
	11	AMC. AMP. IPM. CAZ	1 (2.3)	—	—
	12	AMC. AMP. IPM. CFM	—	—	1 (1.7)
	13	AMC. AMP. CAZ. CFM	3 (7)	—	3 (5.2)

5 ATB	14	AMC. AMP. CN. SXT. NA	1 (2.3)	—	3 (5.2)
	15	AMC. AMP. CN. NA. IPM	1 (2.3)	—	—
	16	AMC. AMP. CN. NA. CAZ	2 (4.7)	—	—
	17	AMC. AMP. CN. SXT. IPM	2 (4.7)	—	1 (1.7)
	18	AMC. AMP. SXT. NA. IPM	—	—	1 (1.7)
	19	AMC. AMP. CN. SXT. CFM	3 (7)	—	—
	20	AMC. AMP. CN. IPM. CFM	—	—	1 (1.7)
	21	AMC. AMP. CN. CAZ. CFM	2 (4.7)	—	4 (6.9)
	22	AMC. AMP. NA. CAZ. CFM	1 (2.3)	—	—
	23	AMC. AMP. NA. IPM. CFM	1 (2.3)	—	—
	24	AMC. AMP. SXT. NA. CFM	2 (4.7)	—	—
	25	AMC. AMP. IPM. CAZ. CFM	—	1 (5.6)	1 (1.7)

6 ATB	26	AMC. AMP. CN. NA. CAZ. CFM	—	1 (5.6)	—
	27	AMC. AMP. CN. NA. IPM. CAZ	—	1 (5.6)	3 (5.2)
	28	AMC. AMP. CN. NA. IPM. CFM	1 (2.3)	—	3 (5.2)
	29	AMC. AMP. CN. SXT. NA. IPM	2 (4.7)	—	3 (5.2)
	30	AMC. AMP. CN. SXT. NA. CAZ	1 (2.3)	—	1 (1.7)
	31	AMC. AMP. CN. SXT. NA. CFM	1 (2.3)	—	2 (3.4)
	32	AMC. AMP. CN. SXT. IPM. CAZ	—	—	1 (1.7)
	33	AMC. AMP. SXT. NA. IPM. CFM	2 (4.7)	—	—
	34	AMC. AMP. NA. IPM. CAZ. CFM	2 (4.7)	—	—
	35	AMC. AMP. CN. IPM. CAZ. CFM	1 (2.3)	—	—
	36	AMC. AMP. SXT. NA. CAZ. CFM	1 (2.3)	1 (5.6)	—
	37	AMC. AMP. SXT. IPM. CAZ. CFM	1 (2.3)	—	—

7ATB	38	AMC. AMP. CN. SXT. NA. IPM. CAZ	4 (9.3)	—	3 (5.2)
	39	AMC. AMP. CN. SXT. NA. IPM. CFM	1 (2.3)	—	1 (1.7)
	40	AMC. AMP. CN. NA. IPM. CAZ. CFM	—	3 (16.7)	1 (1.7)
	41	AMC. AMP. CN. SXT. NA. CAZ. CFM	1 (2.3)	—	2 (3.4)
	42	AMC. AMP. CN. SXT. IPM. CAZ. CFM	—	—	1 (1.7)
	43	AMC. AMP. SXT. NA. IPM. CAZ. CFM	1 (2.3)	1 (5.6)	2 (3.4)
8 ATB	44	AMC. AMP. CN. SXT. NA. IPM. CAZ. CFM	—	9 (50)	8 (13.8)

-^*∗*^: absent; AMP: ampicillin; CN: gentamicin; SXT: trimethoprim-sulfamethoxazole; NA: nalidixic acid; IPM: imipenem; CAZ: ceftazidime; and CFM: cefotaxime.

## Data Availability

All the data used in this study are included within the article.
